# Does Psychological Flexibility Correlate with Mystical Experiences: A Machine Learning Approach Including State of Surrender, Near-Death Experiences, and Psilocybin Consumption

**DOI:** 10.3390/bs16050686

**Published:** 2026-04-30

**Authors:** Dylan Briggs, Thomas B. Sease, Ruthie Menou, David R. Perkins

**Affiliations:** 1 Department of Psychology, University of Louisiana at Lafayette, Lafayette, LA 70504, USA; 2 College of Science and Engineering, Texas Christian University, Fort Worth, TX 76109, USA

**Keywords:** mystical experiences, psychological flexibility, state of surrender, near death experiences, psychedelic use

## Abstract

Mystical experiences are characterized by a profound sense of interconnectedness and transcendence of ordinary reality. These experiences can facilitate feelings of connectedness with oneself and others and have been documented as leading to significant positive changes in thoughts, emotions, and behavior. The purpose of the present study was to evaluate the extent to which the four mindfulness facets of psychological flexibility (i.e., experiential acceptance, present-moment awareness, cognitive defusion, and self-as-context) were associated with self-reported mystical experiences while controlling for established covariates. Using a sample of 150 individuals recruited online, a regularized regression with an elastic net—a computationally efficient machine learning algorithm—was used to model the relationships among mystical experiences, State of Surrender, frequency of psychedelic use, near-death experiences, and facets of psychological flexibility. State of Surrender, experiential acceptance, cognitive defusion, and present-moment awareness emerged as the most robust predictors of mystical experiences. Collectively, these findings underscore the role of psychological processes, including surrender-related processes and facets of psychological flexibility, in predicting mystical experiences.

## 1. Introduction

Several mental health crises are simultaneously occurring worldwide, and have become a noteworthy pandemic, with approximately 800,000 people dying by suicide each year (WHO, 2022), drug and alcohol use being at an all-time high (Pitman et al., 2020), and mental illness afflicting millions (McCracken et al., 2022). Ongoing struggles with mental health have led many people to seek out positive outlets that may alleviate the suffering associated with these mental health challenges. For example, some individuals have focused on how various practices and concepts within the field of psychology may be used to help improve people’s overall life satisfaction and well-being (Bahlinger et al., 2022; McCracken et al., 2022). People struggling with mental illness may seek out life-enhancing experiences, which have led to increased attention to mystical experiences.

### 1.1. Mystical Experiences

Mystical experiences are characterized by a profound sense of interconnectedness and transcendence of ordinary reality (Gandy, 2022). These experiences can facilitate feelings of connectedness with oneself and others as well as a broader sense of awe (Keltner & Piff, 2020; Russ et al., 2019b). According to Sielaff et al. (2022), mystical experiences consist of four main components: mystical transcendence of time, mystical transcendence of space, increased positive mood, and ineffability. Mystical experiences have been documented as leading to significant changes in thoughts, emotions, and behavior (Griffiths et al., 2006, 2008). Moreover, mystical experiences have been positively associated with self-awareness, a sense of meaning, and the achievement of transcendence (Keltner & Piff, 2020). Given mystical experiences’ impact on psychological well-being (Kangaslampi, 2023), a considerable amount of research has been conducted to identify those psychological and behavioral factors thought to precipitate mystical experiences.

### 1.2. Established Behavioral and Psychological Correlates of Mystical Experiences

Mystical experiences can occur through many environmental and behavioral pathways. For example, individuals may feel a sense of oneness with the universe and gain profound insights into their own existence following near-death experiences (Greyson, 2003), religious rituals (Hood, 1975), contemplative practices (de Castro, 2015), and the use of psychedelics (Russ et al., 2019b). Religious rituals may lead to transcendent states of consciousness or encounters with divine beings in some individuals (Greyson, 2014). Additionally, psychedelic substances like psilocybin can induce mystical experiences marked by ego dissolution and unity with the universe (Ko et al., 2022). Overall, these experiences offer unique pathways to altered states of consciousness and spiritual insights that, at times, have been associated with improved psychological well-being (Kangaslampi, 2023).

Undergoing a near-death experience can lead to a mystical experience, with people who undergo an altered state of consciousness during a near-death experience sometimes encountering pervasive changes in how they perceive life around them (Greyson, 2006). Timelessness, transcendence, ineffability, and a sense of divine union are some phenomena that share characteristics of mystical experiences in individuals who have had a near-death experience (Greyson, 2014). An individual’s sudden awareness of life’s limits has been theorized by Hood (1977) as a mechanism that can elicit a feeling of transcendence. Indeed, research has shown a link between near-death experiences and mystical-like experiences (Greyson, 2006; Pennachio, 1986). Depending on the depth of the near-death experience, those impacted by the event often undergo changes characteristic of a spiritual transformation (Greyson, 2006).

Another important factor linked to mystical experiences is individual’s use of contemplative practices. The use of contemplative practices can lead to short-term psychological changes and have a variety of downstream benefits for the individual practicing them (de Castro, 2015). Phenomena that are characteristics of mysticism are included in some of these changes (Büssing et al., 2012). Examples of common contemplative practices discussed throughout the literature include, but are not limited to, exercises such as yoga, prayer, and mindfulness meditation (de Castro, 2015; Jalili, 2024). The Null Directed Methods (NDM) meditation is a meditative technique where the individual is emptied of phenomenological content in an attempt to enter a null and empty state during the meditation (Nash et al., 2013). The NDM meditation technique was found to be associated with individuals’ mystical experience in their daily lives in Palmer et al. (2024). Contemplative practices have also been linked to the intensity of mystical experiences, evident in the study conducted by Qiu and Minda (2023) where participants who had more intense mystical experience and higher mindfulness had predicted their outcomes of wellbeing. Yoga, prayer, and mindfulness meditation were the three contemplative practices observed in a study conducted by de Castro (2015) that focused on the associations between contemplative practices and mystical experiences. In this study, people who reported meditating, while combined with other practices, had higher mindfulness scores when compared to those who did not practice meditation, those who did yoga, or individuals who pray (de Castro, 2015).

Psychedelic substances, such as ketamine, psilocybin, and ayahuasca, have been increasingly examined in relation to mystical experiences (e.g., Ko et al., 2022). Additionally, mystical experiences have been identified as a potential mechanism of change influencing clinical outcomes in the context of psychedelic-assisted therapy (Ko et al., 2022). Psychedelic research has had somewhat of a resurgence (see Payer et al., 2024), with a number of studies showing that psychedelic consumption is associated with mystical-like experiences. For instance, previous research has shown that aspects of psychological flexibility (e.g., acceptance) were significantly correlated with outcomes of mystical experiences in psychedelic assisted therapies (Ko et al., 2022). Additionally, individuals participating in therapies assisted by psychedelics experienced a reduction in anxiety and depression symptoms that were related to their cancer (Luoma et al., 2019). The use of psychedelics has been shown to have a positive impact on emotional health, with some individuals experiencing mental flexibility following the use of psychedelics (Viña, 2025).

State of surrender (SoS) is a psychological state characterized by an individual’s willingness to accept what is to come in their life without resistance. SoS was a concept coined by W. James (1902) and has since been used in studies on psychedelic experiences (Russ & Elliott, 2017; Russ et al., 2019b) and psychological well-being (Sease et al., 2024). SoS is related to psychological flexibility, wellbeing, and mindfulness (Sease et al., 2024; study 2). According to Russ et al. (2019b), one’s ability to be flexible and to release concerns can facilitate mystical experiences, a process thought to be driven by a psychological State of Surrender. A surrender state has identified as a key predictor of mystical experiences (Russ & Elliott, 2017; Russ et al., 2019b), even while controlling for other important covariates, with SoS being the strongest predictor of mystical experiences.

### 1.3. Psychological Flexibility as a Potential Correlate of Mystical Experiences

Psychological flexibility refers to an individual’s ability to adapt and respond effectively to various situations, emotions, and thoughts even in discomfort or distress (Landi et al., 2021). Those who possess higher levels of psychological flexibility are more likely to be in the present moment (Curtiss & Klemanski, 2014). By contrast, psychological inflexibility refers to rigid behaviors that impede an individual’s ability to manage stressful situations and life changes. Those who are psychologically inflexible may struggle with adapting to changes in their life due to being stuck in thoughts, emotions, and behaviors. The literature on this topic will sometimes refer only to the concept of psychological inflexibility (Latzman & Masuda, 2013; Woodruff et al., 2014), only to the concept of psychological flexibility (Dubler, 2018; Watts & Luoma, 2020), or to both as separate but related constructs (Howell & Demuynck, 2021; Qi et al., 2023). Emerging psychometric research and conceptual discussions have argued that these concepts are not necessarily opposites (Rolffs et al., 2018), pointing out that the absence of suffering does not imply the presence of well-being. Since psychological flexibility tends to correlate with positive psychological outcomes (e.g., Marshall & Brockman, 2016; Wersebe et al., 2018)—such as mystical experiences—the current study focused on psychological flexibility rather than inflexibility.

Within the context of Acceptance and Commitment Therapy (ACT), a form of cognitive-behavioral therapy, psychological flexibility involves several core processes. Specifically, psychological flexibility comprises the following six components: experiential acceptance, cognitive defusion, contact with the present moment, self as context, committed action, and values). The goal of developing psychological flexibility is to enhance well-being, reduce suffering, and improve one’s ability to adapt to life’s challenges (Marshall & Brockman, 2016). Psychological flexibility can help individuals better navigate stress, anxiety, depression, and various life transitions, ultimately leading to a more meaningful and fulfilling life (Curtiss & Klemanski, 2014; Dubler, 2018; Landi et al., 2021). Psychological flexibility has been referred to as resiliency (Landi et al., 2021), as well as emotional and mental well-being (Marshall & Brockman, 2016).

Psychological flexibility and mystical experiences may be correlated due to both constructs involving acceptance, openness, awareness in the present moment, and transcendence. In the previous literature, psychological flexibility was associated with mystical experiences (Davis et al., 2020; Krabbe et al., 2024). More specifically, in a study conducted by Davis et al. (2020), mystical experience was positively correlated with participants’ self-reported psychological flexibility. Additionally, a study conducted by Krabbe et al. (2024) found that psychological flexibility and mystical experience were correlated with one another in participants undergoing psychedelic-assisted therapies to treat depression. Psychological flexibility has recently been identified as a target in psilocybin use for treatment of depression and has been associated with ACT (Krabbe et al., 2024). Previous literature has shown that psychological flexibility to have positive associations with mystical experiences (Campo & Yali, 2025; Davis et al., 2020; Krabbe et al., 2024). In an extension of previous efforts, the purpose of the current study was to evaluate the extent to which the components of psychological flexibility were correlated with mystical experiences while controlling for other established correlates.

### 1.4. Present Study

The current study sought to answer the following research question: Do the four mindfulness components of psychological flexibility correlate with mystical experiences while controlling for other environmental and behavioral factors linked to mystical experiences? The current study used a sample of 150 people recruited online to evaluate this research question and model the relationships among mystical experiences, psychological flexibility, and other established correlates (e.g., psychedelic use, near-death experiences, State of Surrender). Additionally, given the conceptual overlap between many of our variables of interest, we used a regularized regression to generate a statistically driven model predicting mystical experiences. Regularized regression is a computationally efficient machine learning algorithm with the advantage of penalizing poor performing predictors and addressing multicollinearity (Boehmke & Greenwell, 2020, Chapter 6), making it a viable alternative to other statistically driven models commonly used in the behavioral sciences (e.g., stepwise regression). Overall, it was expected that the core components of psychological flexibility would correlate with mystical experiences when other variables were controlled for in the model.

## 2. Method

### 2.1. Participants

One hundred sixty-two participants provided informed consent and completed at least some of the research survey. Twelve participants were excluded from the analytic sample for failing to answer most (>50%) of the questions. As illustrated in Table 1, the final sample consisted of 78 (52%) males, 68 (45%) females, and 4 (3%) people who chose not to disclose their gender identity. Most participants were White (n = 109, 73%), followed by Black (n = 19, 13%), Asian (n = 8, 5%), Hispanic or Latino (n = 6, 4%), Native American or American Indian (n = 2, 1%), Pacific Islander (n = 1, 1%), and Other (n = 5, 3%). The age distribution revealed a broad spectrum of participants, with 5 (3%) people between 18 and 24, 60 (40%) between the ages of 25—34, 43 (29%) between 35 and 44, 23 (15%) between 45 and 54, 11 (7%) between 55 and 64, and 8 (5%) aged 65 and older.

### 2.2. Measures

#### 2.2.1. The Mystical Experience Questionnaire (MEQ-30)

The 30-item Mystical Experience Questionnaire (MEQ-30) was used to assess the intensity and depth of mystical or transcendent experiences that individuals may have during various spiritual or psychedelic experiences (Barrett et al., 2015). The scale includes a set of statements designed to capture the critical features of a mystical experience, including unity with the universe, a sense of transcendence, ineffability, a deep understanding of peace, and a feeling of encountering ultimate reality. Respondents are asked to rate their agreement with these statements on a 6-point Likert scale to provide a quantitative measure of the mystical quality of their experience. 0 = “*none; not at all*,” 1 = “*so slight cannot decide*,” 2 = “*slight*,” 3 = “*moderate*,” 4 = “*strong (equivalent in degree to any previous strong experience or expectation of this description)*,” and 5 = “*extreme (more than ever before in my life and stronger than 4)*.” Example items from each subscale of the MEQ-30 includes: “feeling that you experienced eternity or infinity” (mystical subscale), “sense of being ‘outside of time, beyond past and future” (transcendence subscale), “feeling of peace and tranquility” (positive mood subscale), and “sense that the experience cannot be described adequately in words” (ineffability subscale). The MEQ-30 exhibited strong reliability across its four factors, as indicated by Cronbach’s alpha values (α = 0.86–0.97). A higher score means the occurrence of a mystical experience (Barrett et al., 2015).

#### 2.2.2. The Multidimensional Psychological Flexibility Inventory (MPFI)

The Multidimensional Psychological Flexibility Inventory (MPFI) was used to measure psychological flexibility (Landi et al., 2021). The measure provides scores for the six hexaflex areas (i.e., acceptance, defusion, contact with the present moment, self-as context, values, committed action; Rolffs et al., 2018). The responses to these items are measured on a Likert scale, where participants indicate the extent to which they agree or disagree with each statement. Each item was rated on a 6-point scale (1 = *Never True*, 2 = *Rarely True*, 3 = *Occasionally True*, 4 = *Often True*, 5 = *Very Often True*, 6 = *Always True*,). Higher scores suggest greater psychological flexibility, indicating a person’s adaptability, resilience, and ability to navigate challenging situations in accordance with their personal values (Landi et al., 2021; Sundström et al., 2022). The six core measures of psychological flexibility had acceptable internal reliability scores in the current study (αs = 0.96 to 0.97).

#### 2.2.3. The State of Surrender Scale (SoS)

The 8-item State of Surrender scale (SoS) was used to measure an individual’s willingness to accept what is to come without fighting back. Participants were asked to rate their agreement or disagreement with each statement on a 4-point Likert scale (1 = *Strongly Disagree*, 4 = *Strongly Agree*). An example item is: “*I’ve stopped resisting and released control.*” The Cronbach’s alpha for SoS is 0.91. A higher score indicates a higher level of state of surrender, whereas a lower score indicates a lower level of state of surrender (Russ & Elliott, 2017; Russ et al., 2019b).

#### 2.2.4. Procedure

The data for this study were collected using CloudResarch—an online platform that screens high-quality participants from Amazon’s Mechanical Turk. The participants of the study were recruited to participate under the study via Amazon’s Mechanical Turk under a vague title as not to reveal the true nature of the study to prospective participants. The description of the study on the online platform stated that the questionnaire was an academic study on the CloudResearch website, along with providing the participants with the expected time commitment and compensation. Inclusion criteria required participants’ to be at least 18 years of age and above, reside in the United States, and read and understand the English language. After providing informed consent, participants were asked to complete the following questionnaires/scales: MEQ-30 (Barrett et al., 2015), the Multidimensional Psychological Flexibility Inventory (MPFI; Landi et al., 2021), the State of Surrender scale (SoS; Russ & Elliott, 2017), and a demographics form. The order of the scales was randomized, and the demographic form was provided last. In exchange for participation, participants were compensated 1 USD. The procedures of this study were approved by the first author’s Institutional Review Board prior to data collection. Participants were excluded from the study if they did not pass the several attention checks (i.e., “what color is the sky”) that were implemented in the study.

### 2.3. Data Analysis Strategy

Descriptive statistics and frequencies were generated for participants’ background information, scale scores were calculated for all variables of interest, and measures of central tendency and dispersion are displayed in Table 2.

The data were pre-processed by dummy-coding categorical variables and centering and standardizing continuous variables. Then a model predicting mystical experiences was estimated using regularized regression with an elastic net (see Boehmke & Greenwell, 2020, Chapter 6 for a more thorough review)—a computationally efficient machine learning algorithm that automates feature selection.

Unlike ordinary least squared (OLS) regression approaches, elastic net regression applies a penalty when modeling regression coefficients, shrinking less impactful predictors towards zero while retaining those variables that are most impactful. This approach is particularly well-suited in situations where predictor variables are highly correlated (i.e., multicollinearity), as it reduces instability in regression coefficients and identifies a parsimonious final model that best explains the outcome variable of interest (Boehmke & Greenwell, 2020; Zou & Hastie, 2005).

Regularized regression with an elastic net also leverages a penalty parameter (λ) that restrains the magnitude and variability of regression coefficients, thereby mitigating the impact of multicollinearity and nonpredictive model features. In other words, the penalty parameter determines the strength of regularization, with smaller values involving less shrinkage and therefore more complex models. Alternatively, models using a larger penalty parameter produce simpler models by shrinking more coefficients towards zero. The penalty parameter therefore reflects a tradeoff between model fit and parsimony, allowing the researcher to balance interpretability with predictive accuracy. Regularized regression approaches, such as those involving an elastic net, are well-suited for moderate sample sizes, as they reduce overfitting and improve generalizability in the presence of correlated predictors (Boehmke & Greenwell, 2020; G. James et al., 2013).

In this study, a grid search, including 5-fold cross validation, was performed on the data to determine the optimal value of the penalty parameter. This approach evaluates model performance across multiple partitions of the data, selecting the value that minimizes prediction error without sacrificing model stability. When estimating the final model, standardized regression coefficients were used to identify features most strongly related to mystical experiences. All data for this study was pre-processed and analyzed using Rstudio, with the glmnet package (Friedman et al., 2010) being used to estimate the regularized regression model.

The ability to test various combinations of variables is an unique advantage of the present study’s use of the regularized regression model. This statistical model allows for State of Surrender to be combined in various computations which yield the most efficient and robust predictor of the occurrence of mystical experiences in the present study.

## 3. Results

The descriptive statistics and bivariate correlations for all variables of interest are displayed in Table 2. The dependent variable—mystical experiences—was positively associated with State of Surrender (*r* = 0.64), as well as each facet of psychological flexibility (*r*s = 0.43–0.46). In contrast, mystical experience scores were not associated with the frequency of psychedelic use (*r* = 0.08) and did not vary as a function of participants’ history of near-death experiences, *t*(148) = 1.51, *p* = 0.13, *d* = 0.27.

Several psychological flexibility facets were highly correlated (*r*s = 0.62–0.85), suggesting considerable overlap among these constructs and highlighting the need to account for multicollinearity in subsequent analyses. Finally, State of Surrender scores were positively associated with all measures of psychological flexibility (*r*s = 0.43–0.54).

### Regularized Regression

A regularized regression was conducted to identify the most robust predictors of mystical experiences while accounting for the multicollinearity observed among predictor variables. An elastic net model with 5-fold cross-validation was estimated. In summary, this approach allowed for the identification of those predictor variables that contributed to the prediction of mystical experiences after accounting for their shared variance.

Continuous variables were centered and standardized prior to entry into the model, and near-death experience history was dummy coded, with no near-death experience serving as the reference category.

Cross-validation suggested an optimal penalty parameter of λ_min = 2.89. A more conservative penalty (λ_1se = 12.80) yielded identical results (see Table 3). In short, when estimated using the λ_min value the results are representative of the model that minimizes prediction error at the expense of model complexity, whereas the results obtained when using the λ_1se value represents a more conservative model with fewer, more stable predictors.

Specifically, both models retained State of Surrender, experiential acceptance, defusion, and present-moment awareness as meaningful predictors. Lifetime psychedelic use, near-death experiences, and self-as-context did not contribute additional predictive value and were therefore excluded once the penalty parameter was applied.

In the final model, estimated using the λ_min, State of Surrender emerged as the strongest predictor of mystical experiences (β = 16.00), followed by facets of psychological flexibility. Experiential acceptance showed the next largest association (β = 3.92), followed by defusion (β = 1.76) and present-moment awareness (β = 1.62). Importantly, these coefficients reflect penalized estimates and therefore should not be interpreted in the same manner as traditional OLS regression coefficients. Rather, these estimates represent the relative importance of each predictor in contributing to the model’s predictive performance. Collectively, these results suggest that State of Surrender was the most robust predictor of mystical experiences, with aspects of psychological flexibility contributing meaningfully but exhibiting comparatively smaller predictive value.

## 4. Discussion

The results in the present study indicated that State of Surrender, experiential acceptance, defusion, and present-moment awareness emerged as the most robust predictors of mystical experiences. These findings align with prior research demonstrating that surrender-related processes influence the occurrence of mystical experiences. This is particularly seen in psychedelic and intensive contemplative contexts (Gandy, 2022; Russ et al., 2019a, 2019b).

Individuals relinquishing control of the present moment and entering a state of surrender has been shown to increase the probability of the occurrence of mystical experiences, opening those who release control to the transformative nature of this phenomenon (Gleick, 2008; W. James, 1902). SoS has been shown to predict positive change in individuals (Russ et al., 2019a, 2019b) and has been theorized as a necessary event towards an individual relinquishing a burdening feeling of responsibility and control (W. James, 1902).

In contrast, lifetime psychedelic use and near-death experiences did not emerge as robust predictors in the present study. This finding diverges from prior research suggesting that psychedelic use is associated with the occurrence of mystical experiences (Luoma et al., 2019; Ko et al., 2022) and that near-death experiences are linked to mystical phenomena (Greyson, 2006; Pennachio, 1986). For instance, individuals who have reported near-death experiences have previously scored higher on measures of mysticism compared to those without such experiences (Greyson, 2014). The absence of these effects in the current study may reflect the distinction between discrete life events (e.g., psychedelic use, near-death experiences) and enduring psychological processes.

Whereas psychedelic use and near-death experiences represent specific experiential events, psychological flexibility and surrender-related processes may reflect ongoing dispositional tendencies that more consistently predict the occurrence of mystical experiences. In this sense, mystical experiences may be less dependent on particular events and more closely aligned with individuals’ openness to experience and psychological flexibility.

Experiential acceptance, defusion, and present-moment awareness are three of the four mindfulness-based psychological flexibility processes that have been positively associated with mystical experiences in the literature (Davis et al., 2020; Krabbe et al., 2024). These processes reflect core components of psychological flexibility and are conceptually related to surrender-based states that may increase openness to transcendent experiences. While the hexaflex model is composed of several components aimed at increasing psychological flexibility, the State of Surrender may be a more unitary construct with a more central focus on shifting the individual to relinquish control and to readily accept the past in the absence of resistance and struggle (Russ et al., 2019a, 2019b). The central focus of SoS may help to facilitate more robust and predictive connections with the occurrences of mystical experiences in comparison due to the multi-component of the hexaflex model. When investigating both the hexaflex model and SoS, there may not be much overlap with hexaflex in the remaining regressions of the model.

The present findings are also consistent with literature centered on therapeutic approaches and processes of flexibility. Interventions incorporating psychedelics, including ACT-based approaches (Campo & Yali, 2025), have demonstrated improvements in self-acceptance and related flexibility processes. For example, participants who ingested psilocybin scored higher on self-acceptance compared to placebo controls (Smigielski et al., 2019). Defusion and present-moment awareness have likewise been implicated in positive therapeutic outcomes, with reports of increased mental clarity and heightened perceptual awareness following psilocybin administration (Watts & Luoma, 2020). While research regarding psychedelic use and the occurrences of mystical experiences has focused on therapeutic effectiveness, the present study aimed to uncover any robust predictors of the occurrence of mystical experiences. Collectively, these findings support the present results suggesting that surrender-related and psychological flexibility processes are closely tied to mystical experiences.

### Limitations

It is important to consider several limitations of the present study. Regarding the sample size of this study, there was a relatively small participant sample (*n* = 150). The relatively small participant sample is attributable to the use of secondary data analysis based on a thesis dataset which originally collected data in 2024. This limitation could impact the stability and replicability of the current study’s findings. Although the variables retained in the final model have demonstrated associations with mystical experiences in prior research (e.g., Davis et al., 2020; Krabbe et al., 2024; Russ et al., 2019a, 2019b), a larger sample size would likely yield more stable parameter estimates and provide a more precise representation of the relative predictive strength of each variable included in the final model. The participant sample was also predominantly Caucasian, with White individuals accounting for 73% of the sample size. While differences in mystical experiences by demographics is relatively limited, national survey data suggest that Black adults report a higher frequency of spiritual experiences when compared to White adults (Pew Research Center, 2023). Thus, demographic experiences may shape the prevalence or occurrence of mystical-like experiences, making future work that oversamples underrepresented groups a critical step in understanding the generalizability of this study’s findings. In an effort to promote greater diversity among participants in this area of research, future studies should utilized additional recruitment strategies aimed at engaging ethnically diverse populations and ensuring that there is more equitable representation among participants in research centered on the prevalence or occurrence of mystical-like experiences.

The cross-sectional, correlational nature of the present study presents constraints on any definitive conclusions about whether these psychological processes precede or result from mystical experiences. Finally, all variables were assessed via self-report measures, introducing the possibility of shared method variance and increasing the risk that observed associations may be inflated due to common method bias.

There has been little prospective work performed in the area of investigating whether higher levels of psychological flexibility allow for the occurrence of mystical experiences. The present study, based on the conceptual congruence of the variables, assumes that psychological processes (i.e., state of surrender, experiential acceptance, defusion, and present-moment awareness) predicted mystical experiences in our sample. However, it is likely that mystical experiences also increase these flexibility-related processes.

## 5. Conclusions

The present study aimed to identify the situational and psychological processes most strongly associated with participants’ self-reported mystical experiences. Psychological flexibility processes were examined alongside other established correlates using a regularized regression approach that penalizes poorly performing predictors while accounting for multicollinearity. State of Surrender, experiential acceptance, defusion, and present-moment awareness emerged as the most robust predictors of mystical experiences. Taken together, the findings suggest that mystical experiences are more strongly associated with enduring psychological processes—particularly surrender and facets of psychological flexibility—than with discrete life events such as psychedelic use or near-death experiences. In this way, mystical experiences may reflect less the circumstances under which they occur and more the psychological openness and flexibility individuals bring to those experiences.

The present study raises several questions in the psychological flexibility and mystical experience literature that have not yet been fully addressed. While the current findings do not show a link between the use of psychedelics and the occurrences of mystical experiences, the results do show that other potential predictors of mystical experiences (e.g., State of Surrender, experiential acceptance, defusion, and present moment awareness) can show consistency across larger combinations of potential predictor variables.

## Figures and Tables

**Table 1:** Demographic Variables, Near Death Experiences, and Psychedelics.

Variable	*M* (*SD*) or *N* (%)
Gender	
Male	78 (52.0)
Female	68 (45.0)
Prefer Not to Say	4 (3.0)
Race	
White	109 (73.0)
Black	19 (13.0)
Asian	8 (5.0)
Hispanic/Latino	6 (4.0)
Native American/American Indian	2 (1.0)
Pacific Islander	1 (1.0)
Other	5 (3.0)
Age	
18–24	5 (3.0)
25–34	60 (40.0)
35–44	43 (29.0)
45–54	23 (15.0)
55–64	11 (7.0)
65+	8 (5.0)
Near Death Experiences	
Yes	44 (30.0)
No	106 (70.0)
Near Death Experience Types	
No Response	119 (80.0)
Car Accident	10 (7.0)
Medical Condition	10 (7.0)
Plane Crash	1 (1.0)
Physical Accident/Attack	8 (5.0)
Suicide Attempt	1 (1.0)
Psychedelics	
No Psychedelics Consumed	79 (53.0)
LSD	25 (17.0)
Psilocybin	36 (24.0)
Other	10 (7.0)

**Table 2:** Descriptive Statistics and Bivariate Correlation Analyses.

Variable	Mean (*SD*)	1	2	3	4	5	6
1. Mystical Experiences	90.32 (8.22)	--	0.64 *	0.43 *	0.45 *	0.43 *	0.46 *
2. State of Surrender	27.53 (8.22)	0.64 *	--	0.43 *	0.52 *	0.50 *	0.54 *
3. Experiential Acceptance	3.95 (0.98)	0.43 *	0.43 *	--	0.62 *	0.67 *	0.62 *
4. Present Moment	4.51 (1.06)	0.45 *	0.52 *	0.62 *	--	0.76 *	0.66 *
5. Self as Context	4.29 (1.19)	0.43 *	0.50 *	0.67 *	0.76 *	--	0.85 *
6. Cognitive Defusion	4.01 (1.25)	0.46 *	0.54 *	0.62 *	0.66 *	0.85 *	--
7. Lifetime Psychedelic Use	2.38 (1.03)	0.08	0.07	0.08	0.07	0.17 *	0.14

*Note.* * *p* < 0.05.

**Table 3:** Regularized Regression Results.

Variable	Standardized β	Retained at λ_min (2.89)	Retained at λ_1se (12.80)
State of Surrender	16.00	Yes	Yes
Experiential Acceptance	3.92	Yes	Yes
Cognitive Defusion	1.72	Yes	Yes
Present Moment Awareness	1.62	Yes	Yes
Self as Context	0.00	No	No
Psychedelic Use (Frequency)	0.00	No	No
Near Death Experiences (Yes vs. No)	0.00	No	No

*Note.* Continuous variables were centered prior to analyses. The MEQ-30 (i.e., mystical experiences) was the dependent variable in this model. Elastic net regression estimated using 5-fold cross validation. The optimal penalty parameter was λ_min = 2.89. The more conservative penalty was λ_min = 12.80. Coefficients reflect penalized estimates in final model.

## Data Availability

The data in this present study is available upon reasonable request.
